# Burnished Gold Fillings in Soft Cement1Read before the Academy of Dental Science, January 4, 1905.

**Published:** 1905-12

**Authors:** Walter I. Brigham

**Affiliations:** Boston, Mass.


### BURNISHED GOLD FILLINGS IN SOFT CEMENT.[1]

BY DR. WALTER I. BRIGHAM, BOSTON, MASS.

In cement we have a valuable tooth filling. It is as compatible a substance for filling teeth as we have. Many times it meets with failure from the ease of its manipulation, so that it has been neglected, and there is trouble also from the improper preparation of a cavity. There is no filling material which we have, that requires more careful preparation of the cavity than cement. I have many times said myself, and I have heard others say, " We speak of the preserving qualities of a porcelain filling ; in reality a porcelain filling has no preserving qualities whatever, unless you consider the cement that is under the porcelain as a part of the porcelain filling. It is the cement that preserves the teeth, and it is made permanent by being covered with porcelain."

Now, in the method that I am trying to explain, we have just these same conditions. We have a lining of a compatible cement made permanent by covering it with gold. This is not original by any means with me. Many men would say that they have used cement, or cement and gold, and I do not doubt but that they have, but I have never seen it demonstrated in just the way I shall try to, unless perhaps at the hands of Dr. Taylor, of Hartford. He is a man of great resources, and I have great confidence in him. Whatever is said or done by him is surely for the best of his patients, and he was the one that encouraged me to try this method.

Now we do not depend upon the cement to hold the filling, as the filling is locked in just as if the cement was not there. In the case of an incisor where we find considerable breaking down of the tissue. We will imagine that we are looking at the mesial surface of an incisor. The preparation of this cavity is not unlike that of any other cavity, only you are not required to have any definite undercuts. In all of these cases our labial wall is the heavy enamel wall. All these cavities should be opened into from the lingual side, because the decay extends lingually more than labially, and with this method it allows us to save a great deal of the labial wall which we might otherwise have to cut off.

Now, in my method of preparation, I always carry the cervical

---

[1] Read before the Academy of Dental Science, January 4, 1905.

line across nearly straight, because it takes off the vulnerable point at the lingual angle where decay is liable to commence, because if you pack gold from the lingual surface as you should, you have no chances against that wall if curved; if you carry it across straight, you have a chance to pack it against that wall instead of sliding it along. In the preparation of this cavity, we will remove all the decay and the cavity will be retentive. We have, perhaps, a little bit of a recess at cervical border and a little at incisal angle. The cavity, of course, is thoroughly dried out and we have left all of the labial wall that we can. Now by filling that partly full of cement, which is mixed up quite thin, we will attempt to fill it with gold.

In my burnishing method I always use a foil. The gold that I use is No. 4 foil, and it is folded until it is twenty-four thicknesses; taking half of a sheet of gold and folding it until it is twenty-four thicknesses, it would be practically 96 foil. I generally fold it upon a piece of chamois. I fold one-half sheet so it will be three thicknesses. Fold it in the centre and you have six thicknesses. Fold it again and have twelve. Fold that again and you have your gold of twenty-four thicknesses, and that ought to be about 1/8 of an inch wide, and cut off any lengths you choose.

I feel that the gold with the crystalline surface works the best. It seems to me, to remain softer under the burnisher for a longer time than the other forms of gold. We will carry in one or two small pieces with the pliers, force it back into the cement at the cervical border and force the cement over the border. Take two or three pieces and force it under the incisal angle, forcing the cement over the enamel border. Do that while the cement is soft. We can gradually commence and in a minute anchor the two pieces together. And by this time you have forced the cement over all of the borders. Now you keep packing this gold continuously for a long time without disturbing your cement in the least. Do not break off the cement around the periphery for a long time after you think you ought to. Keep packing and burnishing the gold on little by little. After you think you have got near enough to the periphery with gold, if you will take a burnisher and carry it in from this lingual surface, you will break the surface of cement over the walls, and you will be surprised to see how closely you have forced the gold to the periphery.

I do not know how many of you burnish gold, but I imagine that this method of burnishing gold would work much better on the soft cement than a mallet would, although when the cement is hard, I do not see why you cannot mallet it if you choose. If I can claim any originality, it is the fact of keeping your burnisher blued. That is, you want to burnish with a blued surface. If they get bright, gold will stick to the burnisher, and you cannot burnish it well. Heat the gold burnisher red-hot and allow it to cool off.

The cases that you see are the distal surfaces of cuspids. You know that the labial surface of a cuspid has a very strong, heavy enamel wall, and it is liable to decay from the lingual surface and leave that wall intact. It is strong enough to stay for a long time if in any way you can support it. It is in these cases that gold in soft cement works beautifully. Now in a cuspid we would prepare the cavity just as we speak of in the central incisor of allowing the labial wall to remain in place, and filling it practically all from the lingual surface.

A few moments ago I spoke of the necessity of a thorough preparation for the reception of a cement filling, and I will come back in a minute to that. Now you have all seen many times how the enamel deteriorates from the inner surface after it has been in the presence of decayed dentine, and becomes white and chalky. Dentists remove this debris, thinking they have a surface good enough for cement. Supposing we do not remove this chalky substance from the under side of the enamel and allow it to remain and fill it with cement. The result is that pretty soon the cement wears out until it gets to the inner surface of the enamel, and then there is nothing to prevent moisture from getting between the soft deteriorated enamel and cement, working under your filling and leaving a big decayed place. How many times you have removed a cement filling like this, when it was very hard to cut on the surface, but when you got to the bottom of the cavity, it was soft and easily removed. That was because the moisture worked down from improper preparation of the cavity. But if you will take an instrument and clean this soft surface of the enamel or dentine until thoroughly clean and hard, so that when the cement gets against it, it has a tight union and will last a long while. Now we have just this same condition with a porcelain inlay, if you do not remove the deteriorated enamel and dentine wall. If you had porcelain here, after the cement worked out at the joint nothing would prevent it leaking and softening the cement in a little while. You might have the same condition with the gold in cement, if you allowed the edge of the cement to come to the enamel border.

Now we come to the bicuspids, and we use the same method. We fill in with cement, and by taking a few pieces of gold and putting them under the lingual or palatal border and pressing them back so that they will be under the walls, we can gradually anchor these two together. Sometimes we force the cement up over the cervical border, carrying it up slightly. Now we will remove the excess of cement there, and you have all the chance in the world to burnish on your gold and finish the fillings.

These are the burnishers that I use. They are very simple affairs indeed, but meet nearly all cases. There is a bicuspid that was filled, holding them in my hands and burnishing the gold into the soft cement. Then the tooth was ground off to show the anchorage which we had there. The surface of that gold where it was ground off was not burnished afterwards at all, and it was not consolidated in the least. It shows how homogeneous the filling is.

Here is an incisor that was filled the same way and ground off to show the cement underneath. At the present time I am capable of leaving more cement in a bicuspid filling than there is there. That was made a long time ago, and the cement was forced out to a great extent, and yet you can see the line of cement thoroughly around the filling.

I think it is often of advantage to use the matrix. It is a simple matter to burnish gold into a bicuspid with the matrix in place. All that you want to do is to keep that part of the gold which is next to the tooth a little bit higher than that which is next to the matrix.

I have no patience with the ruthless cutting away of a tooth substance for prevention of decay. I do believe in it to a conservative extent, but I doubt if there are many here who realize how it is done in the West. I saw there a cavity in a bicuspid that had never broken through to the occlusal surface that took six or seven sheets of gold to fill when they had it cut away. Perfectly ridiculous! They may say that it is necessary to do that to preserve the teeth, but we know it is not. Here in my method we have a cavity-filling that must hermetically seal because we have a soft, plastic substance next to the tooth surface. I believe in cutting these walls away so that they can be kept clean and contoured to hold the teeth in place, and I know of no way that you can get a contour in gold so well as burnishing it.

Dr. Wedelstædt, of St. Paul, is one of those heroic cutters with whom I have no patience whatever. He is a man who says that lots of times he has taken out fillings that other men have put in that were doing good service simply to have a gold filling there. I could put it in terms a good deal severer than I have. I ran up against him in St. Louis, and I have no respect for that method at all. He says he found that the cement had deteriorated. But what caused him to remove the filling. Cement will last under amalgam or gold indefinitely. A year ago I was called upon to fill the lower second molar mesial surface with amalgam in a tooth that I had filled seventeen years before with amalgam. There was no decay there. The filling had anchored back into the crown and broken off. I filled it again, but the cement after seventeen years was just as I left it.

The method is just the same in the crowns of molars, filling them up with cement and putting in your pieces of gold and forcing it over the walls. Of course the proximal cavities in the molars are practically the same as in the bicuspids. Now in a straight pit fissure where I wanted to put in gold, I would simply fill it with cement, and then take a few pieces of gold and pack them down into the centre of this, forcing the cement up over the walls, and we would have a great supply forced up over. As we force this down continually by forcing it into the centre, that would have the tendency to force the gold out a little bit so that it would be anchored in the cement. By cleaning off the surface we have the gold carried over the walls, and we have it anchored with just a simple layer of gold which permanently fills it. This is an ideal way for filling on the lingual surface of incisors where you have been obliged to remove the pulp.

*Dr. Payne.*—You generally wait until the cement is hard before you begin burnishing your original pieces?

*Dr. Brigham.*—Once in a while I have waited, but generally with light pressure and then with harder pressure it will harden so that you can go right along with the work.

In doing burnishing it is better to keep the instrument warm, and the heat of the instrument would have a tendency to hasten the hardening of the cement, so that you seldom wait for that.

*Dr. Payne.*—Isn't the cohesive property of the gold destroyed by the free liquid of the cement?

*Dr. Brigham.*—It seems generally to work admirably, but if the cement does act on the gold the least bit, it can be pushed off and the cohesion takes place just the same.

I burnish with both the ends and the sides of instruments, but ordinarily on the sides. In burnishing with gold, it is advisable not to cover too much of the gold with the burnisher. Where I do not go along flat with my burnisher, I do not cover so much gold, but I get a perfect adaptation. This is of great advantage in many smaller cavities in incisors where it would take heroic cutting away to get lines of margins which would be out of the zone of decay, but with this you have it as perfect as the tooth was in the first place, and you can fill this so that it will last beautifully.

Take a case of a lateral incisor that may have a great deal of lost tissue on the lingual surface, and it may be at the cervical border not a very deep cavity, but towards the incisal edge there will be a great loss of tissue, and with an overhanging enamel border. Now if this cavity were shallow, I would simply have my undercuts filled until I got down to the centre of the cavity, and put a little soft cement under this labial wall and pack gold in there, and we have strengthened that wall and used it for a retention of that filling.

Now I have described in the best way that I could other than by demonstrating it in the mouth, the way that I would insert gold fillings in soft cement. Now what are its advantages? You do not have to make any definite retention or definite undercuts. You can save walls which you otherwise could not. Burnishing gold on the cement allows you to work on walls that you would not think of working on with a mallet. You have facilitated matters. You have made it easier for your patient. In the burnishing of the gold, you can work with much less room than you can with a mallet. It is seldom that I am called on to spread teeth unless in cases where a great deal of tissue has been lost and the teeth have fallen together.

The ease to the patient is a very important factor. Another thing, you have your cavity lined with a non-conducting material and one of the most compatible materials we have.

*Dr. Payne.*—Some ten or twelve years ago Dr. Clapp brought to the attention of the Academy the method of inserting gold fillings, using soft cement at the base of the cavities ; and showed us a large gold filling which he had placed in a central incisor, without undercutting, using the cement for retention. It required considerable force to remove it, more than would be exerted upon it in the mouth.

During the past few years I have used this method almost exclusively. I find it unnecessary to make undercuts in most cases, never in cavities that have four walls.

The cavity is filled about one-half full of sticky cement, being careful not to smear the edges, and DeTrey's gold is worked into the soft cement and then finished with any gold the operator chooses.

You will notice that the preparation of gold called DeTrey's has one surface very porous. That side is placed upon the soft cement, and the cement will work through or into the gold, giving a very strong adhesion. Weston's cement is used, as it is very sticky and sets quickly.

Large pluggers must be used at first, and when the cement commences to harden finer points can be used for condensing.

It has been of the greatest help to me and saves fully one-third of my time in that branch of my work.

The essayist spoke of heating his burnishers red-hot. I should consider an instrument unfit for use that had been so heated a number of times.

*Dr. Brigham.*—Dr. Payne illustrated his method of gold in cement. Now mine is unlike his. I think I like mine better. He depends upon the adhesiveness of his cement to hold his gold. If you will notice that bicuspid, all the powers that be cannot disturb it, yet it was lined with cement. Now by Dr. Payne's method the gold is held like porcelain fillings, which rely upon the adhesiveness of the cement. Now we do put in a good many porcelain fillings without any retention and they will last, but others will not, and I do not know why some will and some will not. But in the method that I tried to describe we force the gold in the cement and the gold is packed under the undercut.

Dr. Payne spoke of the heating of the burnishers. There is no temper in the burnishers and there was never intended to be, and it is not necessary. When I break one, I step into the laboratory and make another.

I do not know that I have anything else to say, only I feel that I have not done the subject justice. If any of you have a day off, the latchstring of my office is always out, and I should like to demonstrate it in the mouth to show how easily and beautifully it works. I have given several clinics upon this subject, and perhaps some of you may see me some time again.

